# Awareness regarding eye donation among stakeholders in Srikakulam district in South India

**DOI:** 10.1186/1471-2415-14-25

**Published:** 2014-03-06

**Authors:** Venkata Ramana Ronanki, Sethu Sheeladevi, Brinda P Ramachandran, Isabelle Jalbert

**Affiliations:** 1 International Centre for Advancement of Rural Eye Care, L V Prasad Eye Institute, Banjara Hills, Hyderabad, India; 2 University of New South Wales, Sydney, Australia; 3 Ramayamma International Eye Bank, L V Prasad Eye Institute, Hyderabad, India

**Keywords:** Eye donation, Corneal blindness, Community stakeholders, Willingness to donate

## Abstract

**Background:**

There is a huge need for the availability of transplantable donor corneas worldwide to reduce the burden of corneal blindness due to corneal opacity. Voluntary eye donation depends on the awareness levels of various stakeholders in the community. This study aimed to assess the awareness level regarding eye donation among various stakeholders in Srikakulam district in the state of Andhra Pradesh, India.

**Methods:**

355 subjects were selected from the district using multi stage random sampling. A pre tested semi structured questionnaire was used to collect information regarding each individual’s awareness, knowledge, and perception regarding eye donation. Each response was scored individually and a total score was calculated. Univariate and multivariate regression analysis was used to determine the factors associated with willingness towards eye donation and increased awareness levels.

**Results:**

Of the 355 subjects interviewed, 192 (54%) were male and 163 (46%) were female. The mean age of the stakeholders was 35.9 years (SD ±16.1) and all the study subjects were literate. Ninety-three percent of subjects were aware of the concept of eye donation. Knowledge levels were similar among the teaching community and persons engaged in social service, but lower among students (p < 0.05). Among the stakeholders, there was considerable ambiguity regarding whether persons currently wearing spectacles or suffering from a chronic illnesses could donate their eyes. Older age group (p < 0.001), female gender (p < 0.001) and education (p < 0.001) were associated with increased knowledge levels. 82% of the subjects were willing to donate their eyes and this was unaffected by gender or geographical location (rural vs urban).

**Conclusions:**

Awareness levels and willingness to donate eyes are high among the stakeholders in Srikakulam district in India. The services of stakeholders could be utilized, in conjunction with other community based eye donation counselors, to promote awareness regarding eye donation among the general population.

## Background

According to the Andhra Pradesh Eye Disease Study (APEDS) findings, the prevalence of corneal blindness was 0.13% (95% CI: 0.06-0.24) and constituted 9% of all blindness [1]. The APEDS study estimated that 1,200 people per million population are blind (<3/60) from corneal pathology. The prevalence of unilateral blindness due to corneal opacity in low income settings is estimated to be in the range of 5,000 to 20,000 people per million populations. More recently, blindness rates of 3.6% (95% CI: 3.3-3.9) were measured in India using a rapid assessment method, with corneal opacities (including trachoma) accounting for 6.5% of blindness cases, providing a more up-to-date nationally representative estimate of the impact of corneal disease on vision and visual impairment [2]. Based on this, approximately 40 million people were blind in India in 2007 of which more than 2.5 million were blind from corneal disease.

The prevalence of corneal disease varies from country to country as also from one population to another, depending on many factors, such as the availability and general standards of eye care [3].

The annual incidence of corneal ulceration in Madurai District in South India was 113 per 100,000 people [4], 10 times the annual incidence of 11 per 100,000 reported from Olmsted County, Minnesota, in the US [5]. The rampant and unjustified use of topical steroids in cases of red eye leading to corneal superficial infection [6] is an important factor for the high prevalence of corneal blindness in developing countries. Other specific causes of corneal blindness include Trachoma [7], Ocular trauma [8], ulceration [9], childhood corneal blindness [10] and use of traditional eye medicines [10,11]. The use of traditional eye medicines (e g, dried plant materials crushed into powder and dissolved in an aqueous medium; animal/human products such as breast milk, saliva, urine, etc.) is an important risk factor for corneal ulceration and blindness in many developing countries.

Public health prevention programs are the most cost effective means of decreasing the global burden of corneal blindness, because it is difficult to treat corneal blindness once it has occurred. Once a corneal scar develops, surgical management remains the only option for visual rehabilitation. Unfortunately, there is often poor availability of donor tissue. According to the Eye Bank Association of India, the current cornea procurement rate in India is 49,000 per year. It is estimated that a significant proportion of donor corneas are unsuitable for corneal transplantation [12]. Based upon our current ratio of available safe donor eyes, 277,000 donor eyes are needed to perform 100, 000 corneal transplants in a year in India [13].

There is a huge need for donor corneas worldwide to minimize the gap between demand and supply of corneas. A shortage of transplantable corneas is the major problem in reducing blindness due to corneal opacity. In developing countries like India, alternatives to corneal transplantation are being sought due to a lack of adequate donor corneas [14]. Previous reports suggest that increasing the awareness level of the communities regarding eye donation will be useful for increasing eye donation in the community [15]. The barriers to corneal donation reported include religious and cultural beliefs (e.g. significance of eyes), objection from the family members, associated health problem affecting the eye donation, and concerns about disfigurement and mutilation [16]. Possible enablers include a desire to help others and positive donor requestor technique and donor requestor training [16].

Studies aimed at communities at large should include stakeholders from different fields. Stakeholders potentially include but are not limited to donors and their family and community, beneficiaries of corneal donation and their family and community, surgeons, hospital staff, and eye banking facilities staff and the general population [12-27]. However, most previous studies have focused on only single groups such as medical students [17-19] or nursing students [20]. There was no study conducted thus far to include various stakeholders from the same community. There are many key players in the community who can create awareness and influence the attitude of the rest of the community towards better health practices. Hence, this study aimed to study the awareness and perception regarding eye donation among different stakeholders in Srikakulam district, India.

## Methods

This study was conducted in Srikakulam district in the state of Andhra Pradesh in India between 1^st^ July and 30^th^ October 2011. An observational study design was used to collect information from the various stakeholders including Multi Purpose Health Assistant (MPHA) (female) trainee students, teachers working in High Schools, persons engaged in social service and kin of the family members who had earlier donated corneas in the district. Individual participants could only be categorised into a single stakeholder group and all stakeholder categories were mutually exclusive. The list and grouping of potential stakeholders was devised through an iterative process based on the previous literature and in consultation with members of the local community. All stakeholder lists were generated at the district level and organized mandal (an administrative unit) wise. Multi stage random sampling was done to select subjects in each group. For the selection of MPHA and for the persons engaged in social service, only those mandals with presence of the MPHA training centres and the social service organizations were considered. Three schools from each division were selected randomly and the teachers were approached for data collection. The kin of the family members were selected randomly from the list generated from April- 2010 to May 2011 at the district level.

The sample size for this study was estimated based on assuming 40% as pooled awareness among the stakeholders based on published awareness data ranging from 31% [15] to 69% [21] with 6% precision level and 95% confidence level. This indicated that a sample of 256 subjects would be required. Assuming 15% as refusal rate, the total sample required for the study was 300 subjects. In addition, we planned to interview one member from 50 randomly selected families who had previously donated eyes.

The questionnaire was developed based on the previous published reports [15-22] in the area of eye donation. The content, flow and face validity of the instrument was assessed through an iterative process in consultation with community-based experts and upon pilot testing with non-experts. The questionnaire was administered by a single investigator and responses were manually recorded before transferring to a database.

In line with previous work [15-22] a pre-tested, 15 item semi-structured questionnaire was used to obtain information on demographic details, awareness regarding eye donation (4 items), knowledge (6 items), perception (1 item), barriers and enablers to donation (2 items), willingness to donate eyes (1 item) and sources of information (1 item). A copy of the questionnaire is provided in Additional file 1. A separate questionnaire using only open-ended questions was used to sample responses from family of previous donors and is provided in Additional file 2. Informed consent was obtained from all the individuals after explaining the purpose of the study.

The awareness section included basic questions on whether the respondents had heard about eye donation earlier, when does eye donation happen, awareness about prior pledging, and the availability of eye collection centre in the district and whether it causes any disfigurement.

The knowledge section included questions on definition of eye donation, the exact time of removal of cornea, what happens after the eye donation, which part is re-used and for what purpose. The perception section included questions on the eligibility of particular persons for eye donation on the basis of age limit, gender, current use of spectacles, and existence of chronic diseases. Also, this section included their willingness to donate eyes and the reasons for doing so. Answers to question 1 to 13 were scored individually and each right answer was given one score and the maximum possible score for each individual was 13. Answers to question 14 on willingness to donate (see Additional file 1) were scored and categorised as either “yes” or “no/not decided” and no score was given to question 15 related suggestions to improve eye donation in the community.

This study was approved by the Institutional Review Board (IRB) of L V Prasad Eye Institute. The data was analyzed using Epi info, SPSS software and p-values less than 0.05 were considered as statistically significant. Univariate and multivariate regression analysis was used to determine the factors associated to willingness towards eye donation and increased knowledge levels.

## Results

All invited subjects agreed to participate. A total of 355 subjects were interviewed and the mean age of the stakeholders was 35.9 years with a SD of ±16.1 years. Out of 355, 192 (54%) were males and all stakeholders were literate. The demographic details of the study subjects by category are shown in Table 1. Overall, 93% of the respondents were aware of eye donation. Twenty-five subjects (23 students and 2 teachers) reported they had never heard of eye donation earlier.

**Table 1:** Demographic profile of study participants

	**Students n = 124 (%)**	**Teachers n = 105 (%)**	**Persons engaged social service n = 76 (%)**	**Kin of the family members who donated eyes N = 50 (%)**	**Total n = 355 (%)**
**Age**					
<=20 years	82 (66)	0	0 (0)	1 (2)	83 (23)
21-40 years	42 (34)	47 (45)	29 (38)	24 (48)	142 (40)
Above 41 years	0	58 (55)	47 (62)	25 (50)	130 (37)
**Gender**					
Male	0 (0)	70 (67)	76 (100)	46 (92)	192 (54)
Female	124 (100)	35 (33)	4 (8)	4 (8)	163 (46)
**Education**					
Higher secondary	111 (90)	3 (3)	30 (39)	20 (40)	164 (46)
Degree-PG	13 (10)	102 (97)	46 (61)	30 (60)	191 (54)
**Location**					
Rural	70 (56)	95 (90)	51 (67)	33 (66)	249 (70)
Urban	54 (44)	10 (10)	25 (33)	17 (34)	106 (30)

The majority of subjects were aware (80.3%) that eyes could be donated after death. More than 80% of the students, teachers and members engaged in social service and 62% of subjects from the kin of the family member who had earlier donated their eyes believe that prior pledging is essential for eye donation. Except the family members who had previously donated eyes, almost half of the subjects from the community were not aware of the availability of eye collection centres in the district. More than 70% of the subjects across all sectors thought that eye donation does not cause any disfigurement. The awareness and the perception of the subjects in specific reference to age limit, gender, current use of spectacles and persons with chronic conditions are presented in Table 2.

**Table 2:** Knowledge and perception regarding eye donation among the Stakeholders

**Knowledge and perception**	**Students n = 124 (%)**	**Teachers n = 105 (%)**	**Persons engaged in social service n = 76 (%)**	**Kin of the family members n = 50 (%)**	**Total n = 355 (%)**
Heard about eye donation	101 (81.5)	103 (98.1)	76 (100.0)	50 (100.0)	330 (92.9)
Eyes can be donated after death	73 (58.9)	90 (85.7)	72 (94.7)	50 (100.0)	285 (80.3)
Prior permission required for eye donation	97 (78.2)	92 (87.6)	68 (89.5)	31 (62.0)	288 (81.1)
Eye donation doesn’t cause any disfigurement	85 (68.5)	79 (75.2)	55 (72.4)	42 (84.0)	261 (73.5)
Eye donation means removal of cornea	47 (37.9)	61 (58.1)	50 (65.8)	37 (74.0)	195 (54.9)
Ideal time to remove eyes within 6 hours after death	58 (46.8)	74 (70.5)	53 (69.7)	43 (86.0)	230 (64.8)
The donated eyes used to replace cornea for another eye	48 (38.7)	64 (60.9)	56 (73.7)	32 (64.0)	200 (56.3)
Donated eyes can give sight to a blind person	98 (79.0)	99 (94.3)	74 (97.4)	43 (86.0)	314 (88.5)
Availability of nearest eye collection center	59 (47.6)	55 (52.4)	48 (63.2)	42 (84.0)	204 (57.5)
Perception of stakeholders on who can donate eyes					
Person of any age	71 (57.3)	97 (92.4)	73 (96.1)	46 (92.0)	287 (80.8)
All gender	101 (81.5)	102 (97.1)	76 (100.0)	50 (100.0)	329 (92.7)
Current spectacle users	19 (15.3)	62 (59.0)	44 (57.9)	36 (72.0)	161 (45.4)
People with chronic diseases	23 (18.5)	32 (30.5)	26 (34.2)	18 (36.0)	99 (27.9)

The major source of information on eye donation was the mass media with 61% (n = 200) across all the stakeholders followed by information through the eye care professional working in the area with 24% (n = 79). Around 291 (82%) expressed willingness to donate their eyes and 39 (11%) persons expressed unwillingness to donate eyes, while the remaining 16 (5%) were unable to decide about their willingness at the time of interview.

We looked at the association of various factors with the willingness to donate eyes using logistic regression and found that persons of older age were 7 times more willing to donate eyes compared to students, with (P = 0.018, 95% CI (1.39, 34.7)). There was no significant difference found between genders and persons living in urban or rural areas (Table 3). The reasons listed for unwillingness to donate eyes included objection by family members (12(3.4%)), dislike of separating eyes from the body (3(0.8%)), health problems (10 (2.8%)), religious restriction (2 (0.6%)) and family members being unwilling (10 (2.8%)).

**Table 3:** Factors associated with willingness to donate eyes

	**Total (n = 355)**	**Odds ratio (95% confidential interval)**	**P-Value**
**Age (years)**			
<=20	83	1.00	
21-40	142	6.226 (0.88, 44.14)	0.067
41-60	99	6.960 (1.39, 34.79)	0.018
60+	31	4.505 (0.91, 22.30)	0.065
**Gender**			
Male	192	1.00	
Female	163	1.09 (0.45, 2.66)	0.835
**Education**			
Higher secondary	164	1.00	
Degree-PG	191	0.846 (0.34, 2.12)	0.721
**Geographic location**			
Rural	249	1.00	
Urban	106	1.399 (0.68, 2.87)	0.36
**Stakeholders**			
Students	124	1.00	
Teachers	105	0.577 (0.11, 3.07)	0.519
Persons engaged in social service	76	0.160 (0.05, 0.53)	0.003
Kin of the family members	50	1.301 (0.26, 6.48)	0.748

There exists a significant difference of knowledge scores between students and teachers with means 6.74 ± 3.5 vs 9.1 ± 2.1 (p < 0.001), students and persons engaged in social service with means 6.74 ± 3.5 vs 9.6 ± 2 (p < 0.001), students and kin of the family members with means 6.74 ± 3.5vs10.1 ± 2.1 (p < 0.001). The mean knowledge score of all the subjects was 8.53. Knowledge levels were similar in all stakeholders except the student community. In univariate analysis, increasing age (p < 0.001), gender (p < 0.001) and education (p < 0.001) were significantly associated with increased knowledge level.

We interviewed 50 persons who were the next of kin of the family that had previously donated eyes. It was found that 82% (n = 41) of them donated eyes without any prior pledging. In 42 out of 50 eye donations, family members had taken the initiative to donate eyes. The reasons for donating eyes reported by them included “donation is good work”, “it gives sight to other persons” and “useful for others” by 38%, 36% and 18% respectively. Eight percent of them expressed that they were inspired by others who donated the eyes of their family members. Even among the family that had donated eyes earlier only 10% of the rest of the family members have pledged their eyes and the majority felt that pledging is not necessary for eye donation.

## Discussion

This study aimed to address some of the limitations of previous work by involving a wide range of stakeholders in a survey of perception and awareness of eye donation. Our findings validate this approach by highlighting differences in knowledge and awareness in various stakeholder groups. For example, students more frequently believed that age and spectacle use could impact ability to donate. Similarly, persons in social service were less willing to donate than other stakeholder groups. Future advocacy and/or publicity campaigns should be specifically targeted to address these issues.

We found that awareness regarding eye donation among stakeholders was 93% and the willingness to donate eyes was 291 (82%) among them. Interestingly, awareness tended to be lowest amongst the students compared to other stakeholder groups, yet this did not appear to impact willingness to eye donation. This suggests that increased awareness or in fact increased knowledge may not necessarily translate into increased willingness and that perhaps other unmeasured factors may be coming into play.

The major source of awareness information was received through mass media followed by eye care professionals. There are a lot of misconceptions among the stakeholders regarding eye donation. Only half of them were aware that the current spectacles users can donate eyes and only one third were aware that people with chronic diseases can donate eyes. This is an important issue that can be targeted in future campaigns meant to create awareness.

Awareness regarding the availability of eye bank/eye collection centres in the district was found to be low at 57.5%. Although the majority of the stakeholders had awareness and willingness towards eye donation, 43% of them were unaware of the availability of eye collection centers. This could be a major barrier to donation in the community.

Interestingly, 82% (n = 42) of the previous eyes donors had not pledged earlier for eye donation. This demonstrates that pledging is not influenced by the actual donation and that a majority of donations were initiated by the family members belonging to the same community.

In our study, the most common reason for donating corneas was that it gives sight to other blind person. Similar responses were reported from the previous studies conducted in Bangalore [20] and from Malaysia [21]. However, the result from the study done in Toronto reported the main reasons as personal experiences with cornea donation and transplants, and good results from corneal transplant operation [22].

In our study 82% of stakeholders, aged 16–84, were willing to donate their corneas compared with 67% (n = 544) aged 21–65 years in Singapore [22], 34%(n = 276) aged between 18–75 years in Melka, Malaysia [21], 59% (n = 216) in NSW, Australia [23], 41.5% in Delhi, 52% (n = 1039) adults aged 18–99 in north western India [24] and 44.9% in adults aged above 15 in Hyderabad, India [12]. Only our study and another study conducted in Bangalore among students of Nursing showed over 80% as being willing to donate their eyes (n = 182), aged between 18–21 years [21]. These differences can be attributed to high social engagement in our subjects because of their professional relationships i.e. affiliation to an NGO or school, or family member who had donated eyes. Donor corneas in Srikakulam District are harvested using the in situ excision method. This method of collection, which has previously been suggested to be more acceptable than whole globe enucleation [14], may have increased willingness to donate in our study, but it was not designed to measure this.

Our study reported awareness level among the stakeholders as high, 93% (n = 330) compared to previous studies where the reported levels in Singapore and Malaysian population was 69% (n = 276) and 81%, 86% (n = 344) respectively. Similarly, studies conducted in India from Delhi (55.4%),Tamil Nadu (50.7%) [25], and rural Andhra Pradesh (32.9%) [15] were comparatively very low except the study conducted among the students in Bangalore which reported awareness level as 97%. Differences can be attributed to high level of literacy in this sample as well as the reasons discussed in the paragraph above.

In this study, although the awareness levels are high, the willingness to donate eyes was lesser by 10%. A previous study by Tandon reported that prior knowledge of eye donation, literacy and socio economic status didn’t influence the willingness for eye donation and the major reasons for not donating eyes include refusal to discuss the issue and dissuasion by distant relatives, legal problems, and religious beliefs [26]. Previous study from Southern India among rural population reported that females were less willing to donate eyes even though they were more aware of eye donation [15] whereas, in our study females were no less willing than males to donate eyes.

Even though there is increased awareness, the place to approach potential donors and how to enroll them as donors remained a major challenge. A major suggestion from the community to address this issue was prominent display boards at the existing eye collection centers and at the community gathering places. There should be a concerted effort in the community by all agencies, including the Government and private organizations, in creating catalysts at the community level to act as grief counselors to promote eye donation in the community as suggested by Gogate et al. [27].

Ours is the first study that assessed the awareness level among the various stakeholders representing the same community, and population based observational data collection is the advantage of this study. We interviewed only those who were available at the time of data collection and reported their individual perception. Although they are representative samples from the area, the opinions expressed are by individuals and cannot be extrapolated to the rest of the population.

## Conclusion

There is a need to conduct more research to understand how can we motivate and utilize the services of stakeholders in improving the eye donation activities and an in depth study among the kin of the family members who had donated eyes earlier, which is also essential to document the perception. Further research is required to convert the high willingness to donate eyes to increase the actual eye donation among the communities.

## Competing interests

All the authors have no competing financial or non financial interests.

## Authors’ contributions

VR contributed to the design, data collection and analysis. SS contributed to the design, final analysis and written the manuscript. BR contributed to the design and commented on the drafts. IJ contributed to the design and commented on the drafts. All authors read and approved the final manuscript.

## Authors’ information

^1^Student, Masters in Community Eye Health program, ^2^Visiting Adjunct Lecturer, School of Optometry & Vision Science, UNSW and Associate Public Health Specialist, L V Prasad Eye Institute, ^3^Assistant Director, Ramayamma International Eye Bank, L V Prasad Eye Institute, ^4^Senior Lecturer, School of Optometry & Vision Science, UNSW.

## Pre-publication history

The pre-publication history for this paper can be accessed here:

http://www.biomedcentral.com/1471-2415/14/25/prepub

## Supplementary Material

Additional file 1 Awareness and Perception on Eye Donation among Stake Holders in the State of Andhrapradesh. Click here for file

Additional file 2 Perception of the kin of the family members who donated eyes earlier. Click here for file
