# Supplementary material for: Awareness regarding eye donation among stakeholders in Srikakulam district in South India
Source: BMC Ophthalmol. 2014 Mar 6;14:25. doi: 10.1186/1471-2415-14-25 (PMC4015916; doi:10.1186/1471-2415-14-25)
Supplement: Additional file 1 — Awareness and Perception on Eye Donation among Stake Holders in the State of Andhrapradesh. [file 1471-2415-14-25-S1.doc]

**Additional file 1**

**Awareness and Perception on Eye Donation among Stake Holders in the State of Andhrapradesh**

**Demographic Data**

A: Interviewers Name:

B: Date of Interview:

C: Subject No:

D.Name of the respondent:

E: Age:

F: Sex: Male Female

G: Address:

H: Phone no:

I: Education:

1. 5^th^ class
2. 10^th^
3. Inter
4. Degree
5. PG

J. Occupation:

1. Retired Govt. Employee
2. Business
3. School teacher
4. Student
5. Pvt. Employee
6. Others

**Awareness and perceptions**

1. Have you heard of eye donation**?**

1. Yes 2. No

1a. If yes what is eye donation?

1. Removal of eye 2. Removal of cornea 3.don’t know

2. When the eye should be donated?

1. Before death 2. After death

3. When the eyes to be removed from the body?

1. within 3 hrs. 2. within 6 hrs. 3. 6-10 hrs.

4. 24 hrs. 5. Don’t know

4. Who can donate eyes? Age limit / all gender Yes / No / Not known

5. Who can donate eyes? People already wearing spectacles Yes / No / Not known

6. Who can donate eyes? People with chronic diseases Yes / No/ Not known

7. Which part of the eye is re used from the donated eyes?

1. Cornea 2. Conjunctiva. 3. Total eye 4. Don’t know

8. Does prior pledging is essential for eye donation?

1. Yes 2. No

9. How did you come to know about eye donation?

1. through an eye care professional

2. Mass media

3. Person who already committed to donate eye

4. Family members

5. Other sources

6. Multiple answers

10. Do you aware of any eye bank/ eye collection centre at Srikakulam?

1. Yes 2. No

11. Do you know the donated eyes can give sight to a blind person?

1. Yes 2. No. 3. Don’t know

12. Does eye donation cause any disfigurement in the face?

1. Yes 2.No. 3. Don’t know

13. How the donated eyes are used for?

1. To replace another eye

2. To replace a part of another eye

3. To replace the cornea of another eye

4. Research purpose

5. Don’t know

14. Are you willing to donate your eyes?

1. Yes 2. No 3. Not decided

14. (a) If yes, why ?

**1.** Eye donation is a Nobel work

2. Pleasure to help blind

3. Donated eyes can give vision to a person

4. Influenced after reading an article

5. A friend or relative has donated an eye

6. A friend or relative has received a donated eye

7. Influenced by any lecture

8. Other reasons

14. (b) Perceived reasons for not donating eyes:

1. Lack of awareness:

2. Objection by family members

3. Dislike of separating eyes from body

4. Un suitability to donate because of health problems

5. Religious restriction in separating eyes from the body

6. I will be born as blind in my next birth

7. Disfigurement of the face

8. My family members will not be interested to donate after my death

9. Other reasons

15. What is your suggestion to promote eye donation in the community?
