# Supplementary material for: Awareness regarding eye donation among stakeholders in Srikakulam district in South India
Source: BMC Ophthalmol. 2014 Mar 6;14:25. doi: 10.1186/1471-2415-14-25 (PMC4015916; doi:10.1186/1471-2415-14-25)
Supplement: Additional file 2 — Perception of the kin of the family members who donated eyes earlier. [file 1471-2415-14-25-S2.doc]

**Additional file 2**

**Perception of the kin of the family members who donated eyes earlier**

1. Name of the Donor:
2. Gender: Male : Female:
3. Age :
4. Relationship with Donor:
5. What are the causes for death?

1. Natural death 2.accidental death 3.diseased

1. Did the person whose eyes were donated, pledged earlier?
2. Who initiated the eye donation?

1. Why did you initiate eye donation?
2. How many members in your family have pledged for eye donation?
3. Which organization collected the eyes?
4. What was your feeling, when you initiated eye donation?

12. Were you satisfied with the services provided by the organization?
